# Retraction Note: Downregulation of lncRNA ZNF582-AS1 due to DNA hypermethylation promotes clear cell renal cell carcinoma growth and metastasis by regulating the N(6)- methyladenosine modification of MT-RNR1

**DOI:** 10.1186/s13046-023-02719-9

**Published:** 2023-06-06

**Authors:** Wuping Yang, Kenan Zhang, Lei Li, Yawei Xu, Kaifang Ma, Haibiao Xie, Jingcheng Zhou, Lin Cai, Yanqing Gong, Kan Gong

**Affiliations:** 1 Department of Urology, Peking University First Hospital, No. 8, Xishiku Street, Xicheng District, Beijing, 100034 China; 2 Hereditary Kidney Cancer Research Center, Peking University First Hospital, No. 8, Xishiku Street, Xicheng District, Beijing, 100034 China; 3 Institute of Urology, Peking University, Beijing, 100034 P. R. China; 4 National Urological Cancer Center, Beijing, 100034 P. R. China

**Retraction Note:** ***J Exp Clin Cancer Res*** **40, 92 (2021)**

10.1186/s13046-021-01889-8

The Editor in Chief has retracted this article. After publication, concerns were raised about the following issues:

• OSRC2-ZNF582-AS1 CON panel in Fig. 4c appears to partially overlap with the OSRC2-MT-RNR1 OE panel in Fig. 9g;

• The highlighted sequences in Fig. 6g do not appear to correspond to the positions of the peaks as reported in Fig. 6a;

• There appears to be substantial overlap between this paper and two previously-published papers [1] and [2] that have one and two authors in common with this paper, respectively.

• The reference number of the ethics permit as reported in the article appears to have been used in multiple other studies dealing with topics and studies that appear to require separate approvals.

The authors provided an explanation which did not address the concerns adequately. The Editor in Chief, therefore, has lost confidence in the integrity of the article’s findings. The corresponding author has stated on behalf of all authors that they disagree to this retraction.
